# Mung Bean Functional Protein Enhances Endothelial Function via Antioxidant Activity and Inflammation Modulation in Middle-Aged Adults: A Randomized Double-Blind Trial

**DOI:** 10.3390/foods13213427

**Published:** 2024-10-28

**Authors:** Supaporn Muchimapura, Wipawee Thukhammee, Sophida Phuthong, Prapassorn Potue, Juthamas Khamseekaew, Terdthai Tong-un, Weerapon Sangartit

**Affiliations:** 1 Department of Physiology, Faculty of Medicine, Khon Kaen University, Khon Kaen 40002, Thailand; supmuc@kku.ac.th (S.M.); meewep@gmail.com (W.T.); sophiph@kku.ac.th (S.P.); prappo@kku.ac.th (P.P.); juthakh@kku.ac.th (J.K.); terdthai@kku.ac.th (T.T.-u.); 2 Human High Performance and Health Promotion Research Institute, Khon Kaen University, Khon Kaen 40002, Thailand

**Keywords:** mung bean, functional protein, endothelial function, antioxidant enzymes activity, flow-mediated dilation

## Abstract

This study examines the impact of mung bean (*Vigna radiata*) protein consumption on endothelial function in middle-aged adults, focusing on antioxidant enzyme activity and anti-inflammatory markers. Mung beans have shown promise in enhancing cardiovascular function, lowering blood pressure, and improving lipid profiles, but the underlying mechanisms of these functions remain unclear. Conducted as a three-arm randomized, double-blind, placebo-controlled trial, this study involved male and female participants aged 45 to 60 and assigned them to consume either a placebo or a mung bean functional drink containing 10 or 15 g of mung bean protein daily for six weeks. Vasodilation was assessed using flow-mediated dilation (FMD), and oxidative stress markers, antioxidant enzyme activity, and inflammatory markers were measured at baseline and after the intervention. The results indicate that six weeks of mung bean consumption significantly benefits healthy middle-aged adults by enhancing antioxidant enzyme activity and reducing inflammatory mediators’ expression. Additionally, the increase in brachial artery diameter following FMD indicates improved endothelial function.

## 1. Introduction

Cardiovascular disease (CVD) is a major health concern for middle-aged adults, influenced by factors such as physical inactivity, malnutrition, and chronic conditions like diabetes and obesity [1]. Oxidative stress significantly contributes to CVD by causing endothelial dysfunction, which impairs the ability of blood vessels to dilate and regulate blood flow [2,3,4]. This endothelial dysfunction is a critical early event in the development of cardiovascular complications.

Oxidative stress exacerbates the depletion of endothelial nitric oxide (NO) availability and promotes vascular inflammation [5]. Cyclooxygenase-2 (COX-2) plays a crucial role in this process by driving inflammation and thromboxane production, leading to vasoconstriction and atherogenesis. Overexpression of COX-2 enhances the expression of intercellular adhesion molecule-1 (ICAM-1) and vascular cell adhesion molecule-1 (VCAM-1), facilitating the recruitment of inflammatory cells to the vascular wall, further accelerating the development of atherosclerosis [6]. Similarly, excessive angiotensin-converting enzyme (ACE) activity promotes vascular remodeling, high blood pressure, and chronic vascular inflammation by raising the levels of angiotensin II (Ang II) [7,8]. Elevated interleukin-6 (IL-6) and activated nuclear factor-kappa B (NF-κB) alter vascular homeostasis by increasing vascular permeability, enhancing monocyte adhesion, and promoting smooth muscle cell proliferation, contributing to endothelial dysfunction and plaque formation. Together, oxidative stress, inflammation, and these molecular pathways heighten the risk of CVD [9].

Essential amino acids (EAAs) play a crucial role in antioxidant activity, anti-inflammatory responses, and reactive oxygen species (ROS) scavenging. They serve as precursors for bioactive compounds like glutathione [10]. Certain EAAs, such as arginine, support nitric oxide (NO) production, which helps regulate redox balance. EAAs also scavenge ROS by promoting the synthesis of antioxidant enzymes like superoxide dismutase (SOD) and catalase [11], further enhancing cellular defenses. Additionally, EAAs modulate the NF-κB pathway, reducing its activity and suppressing the expression of pro-inflammatory genes, including TNF-α, IL-6, COX-2, and VCAM-1, thereby promoting a balanced inflammatory state [12,13]. Through these mechanisms, EAA supplementation could reduce the risk of cardiovascular conditions.

Plant-based foods, such as soy and rice, and dairy-based foods, like whey, have been shown to enhance vascular health due to their high-quality proteins rich in EAAs [14]. Notably, whey protein and its bioactive peptides can improve vascular function by positively affecting blood pressure, arterial stiffness, NO production, and inflammation. Moreover, protein supplementation has been linked to increased antioxidant capacity [15].

Mung bean (*Vigna radiata*, MB) is a nutrient-dense legume known for its rapid growth and adaptability, making it a valuable source of plant-based proteins rich in EEAs, in line with the Food and Agriculture Organization (FAO) recommendations. In addition to its high amino acid content, MB provides dietary fiber, vitamins, and minerals, contributing to its status as a dietary staple worldwide [16]. Recent research highlights the cardiovascular benefits of MB consumption, demonstrating improvements in cardiovascular function, reduced blood pressure, and enhanced lipid profiles, which lower the risk of CVD [17,18]. In addition, MB is also rich in antioxidants, including phenolic compounds such as flavonoids, which help combat oxidative stress and inflammation [19]. With its impressive nutritional profile and health-promoting properties, MB is increasingly recognized as a functional food that supports overall well-being.

This study aims to elucidate the underlying mechanisms by which MB affects cardiovascular health in middle-aged adults. Participants were assigned to consume either MB-derived functional protein or a placebo for a duration of six weeks. Endothelial function was assessed using flow-mediated dilation (FMD) through ultrasonographic imaging. The principle of FMD involves measuring the dilation of the brachial artery in response to increased blood flow after occlusion, reflecting the ability of the endothelium to mediate vascular relaxation. Additionally, oxidative stress markers, antioxidant enzyme levels, and inflammatory markers were evaluated at baseline and at the end of the consumption period. We hypothesized that MB protein consumption would improve vascular dilation, reduce oxidative stress, and lower inflammatory markers compared to the placebo.

## 2. Materials and Methods

### 2.1. Preparation of a Functional Protein Powder Derived from MB

Functional protein was extracted using a previously established method [20]. The process began under alkaline conditions with 2 N NaOH, with the mixture stirred at 1000 rpm for 2 h using a stirrer (RW20, IKA®, Baden-Württemberg, Germany). The resulting solution underwent centrifugation at 8000× *g* for 20 min at 4 °C to collect the supernatant. To precipitate the protein, the pH was adjusted to 4.5 using 2 N HCl, followed by 1 h of stirring and a second centrifugation at 8000× *g* for 20 min to yield a concentrated protein pellet. This pellet was then resuspended in distilled water at a 1:10 ratio, and the pH was adjusted to 7.0 with 2 N NaOH. After stirring for 1 h, the solution was centrifuged again, and 5% maltodextrin was incorporated to ensure a homogenous mixture, preparing it for subsequent spray drying.

### 2.2. Determination of the Total Phenolic Content of a Functional Protein Powder Derived from MB

The total phenolic content was assessed through the Folin–Ciocalteu method. In summary, samples were combined with the Folin–Ciocalteu reagent in a 96-well plate. Following an 8 min incubation, a 7.5% sodium carbonate (Na_2_CO_3_) solution was introduced, and the mixture was left to incubate for 2 h in the dark at room temperature. Absorbance was recorded at 765 nm using a microplate reader, and the results were expressed as milligrams of gallic acid equivalents (GAE) per gram of sample (mg GAE/g) [20].

### 2.3. Determination of Total Flavonoid Content of a Functional Protein Powder Derived from MB

The total flavonoid content was assessed through a colorimetric method [20]. In a 96-well plate, the samples were combined with 2% aluminum chloride and incubated at room temperature for one hour. The absorbance was then measured at 415 nm using a microplate reader, with quercetin serving as the standard reference. The results were reported as milligrams of quercetin equivalent (QE) per gram of sample (mg QE/g).

### 2.4. Determination of the Amino Acid Profile of a Functional Protein Powder Derived from MB

One hundred grams of a functional protein powder derived from mung bean were sent to analyze and report the amino acid profile by Central Laboratory (Thailand) Co., Ltd., (Khon Kaen branch, Khon Kaen, Thailand), based on the high-performance liquid chromatography (HPLC) method.

### 2.5. Determination of Antioxidant Activity of MB

#### 2.5.1. 1,1-Diphenyl-2-Picrylhydrazyl (DPPH) Radical Scavenging Activity

A DPPH solution was prepared in methanol and mixed with 20 μL of the sample at concentrations ranging from 5 to 1000 μg/mL. This mixture was incubated at room temperature, protected from light, for 30 min. Following incubation, the absorbance was measured at 517 nm using a microplate reader. The results were reported as the half maximal inhibitory concentration (IC50) in mg/mL and the percentage inhibition of DPPH radical formation [21].

#### 2.5.2. 2,2′-Azino-Bis(3-Ethylbenzthiazoline-6-Sulfonic Acid) (ABTS) Radical Scavenging Activity

The ABTS radical scavenging assay was performed following previously published protocols and using a mung bean protein extract [21]. Briefly, a working solution was prepared by mixing 2.45 mM potassium persulfate (K_2_S_2_O_8_) and 7 mM ABTS in a 3:2 volume ratio, which was then diluted with deionized water at a 1:20 ratio. A total of 20 μL of the sample, prepared at various concentrations, was combined with 40 μL of distilled water and 150 μL of the ABTS solution. The absorbance was measured at 734 nm using a microplate reader. Trolox (Sigma-Aldrich, St. Louis, MO, USA) served as the standard reference. The results were expressed as IC50 values and the percentage inhibition of ABTS radical formation.

### 2.6. Determination of Anti-Inflammatory Activity via Cyclooxygenase-2 (COX-2) Inhibition by MB

COX-2 activity was assessed using a colorimetric method following the manufacturer’s protocol of the assay kit (Cayman Chemical, Ann Arbor, MI, USA). The reaction mixture comprised 150 μL of assay buffer, 10 μL of MB protein extracts, 10 μL of heme, 10 μL of COX-2 working solution, 20 μL of 10 μM TMPD (N,N,N′,N′-tetramethyl-p-phenylenediamine dihydrochloride) (Sigma-Aldrich, St. Louis, MO, USA), and 20 μL of 100 μM arachidonic acid. This mixture was added to 96-well microtiter plates and incubated at room temperature for 30 min. Absorbance was measured at 590 nm, and the results were expressed as the IC50 value, with indomethacin serving as a reference compound.

### 2.7. Determination of ACE Inhibition by MB

ACE activity was assessed using a previously described assay [20]. Briefly, ACE sourced from rabbit lungs (Sigma-Aldrich, Saint Louis, MO, USA) served as the enzyme source. An aliquot of ACE (0.05 units/mL, 10 μL) was mixed with various concentrations of the sample extract (20 μL) to create experimental samples, while a control sample was prepared by mixing ACE with 5 mM phosphate buffer. Captopril was included as a positive control. Following this, 50 μL of 100 mM Hip-Gly-Gly was added to the reaction mixture and incubated at 37 °C for 35 min. The reaction was halted by adding 120 μL of 3 M sodium tungstate (Sigma, USA) and 0.5 M sulfuric acid, followed by centrifugation at 2500 rpm for 10 min. After centrifugation, an aliquot of the supernatant was transferred to a 96-well microtiter plate, mixed with 20 μL of 60 mM 2,4,6-trinitrobenzenesulfonic acid (TNBS), and incubated in the dark for 20 min. Absorbance was then recorded at 415 nm using a microplate reader, with results expressed as the IC50 value.

### 2.8. Study Design

**Figure 1:**
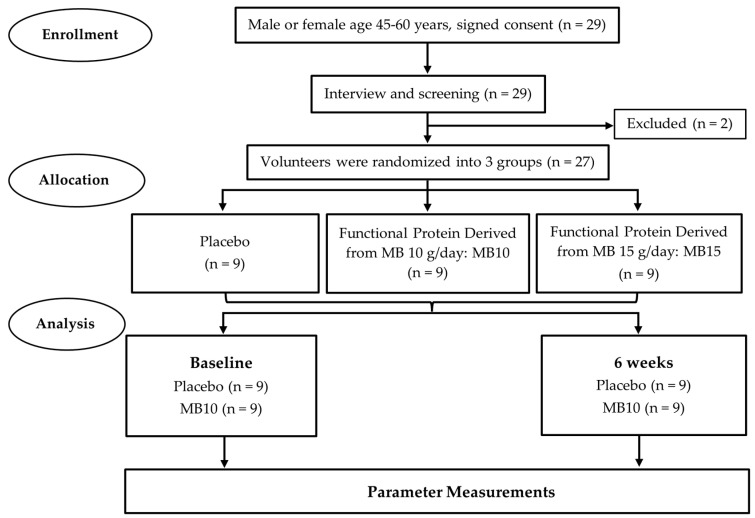
A flow diagram for the study trial.

This study was a 6-week, three-arm, randomized, double-blind, placebo-controlled parallel group trial conducted at the Faculty of Medicine of Khon Kaen University, Thailand. The aim was to evaluate whether MB-derived protein could serve as a functional supplement for improving endothelial function in healthy middle-aged individuals. The research adhered to the guidelines outlined in the Declaration of Helsinki, and all procedures involving human subjects were approved by the Institutional Review Board of the Khon Kaen University Ethics Committee for Human Research, Khon Kaen, Thailand (HE661265). The study protocol was also registered with the Thai Clinical Trials Registry (TCTR20230610002). A total of 29 healthy middle-aged individuals (ages 45–60 years) from the Khon Kaen province, Thailand, were recruited for this study after signing consent forms. Advertisements were placed in the local community, targeting participants with a body mass index (BMI) between 18 and 25 kg/m². After screening for eligibility through a semi-structured interview and physical examination, two subjects were excluded due to their inexperience with dietary interventions, which could potentially affect their adherence to the study protocols and impact the validity of the results. Inclusion criteria required participants to be healthy men and women within the specified age range. Individuals with severe underlying conditions that require continuous medication, such as cardiovascular disease, respiratory disease, diabetes, high blood pressure, kidney disease, and liver disease, were excluded, along with smokers of more than 10 cigarettes per day, those with alcohol addiction, athletes who exercised more than three times a week, participants in other studies, and those taking additional supplements. Eligible subjects were randomly divided into three groups: (1) placebo, (2) MB10, and (3) MB15. The MB10 and MB15 groups received a functional drink containing 25 g of product per serving, with doses of 10 or 15 g of MB-derived protein, respectively. The placebo drink contained the same ingredients as the functional drink but lacked the MB-derived protein. Both the placebo and functional drinks had an identical appearance and taste. Throughout the study, no subjects withdrew. Ultimately, 27 participants completed the 6-week study period, as shown in Figure 1. Subjects were instructed to mix the functional drink powder with water and consume it daily before meals for the duration of the study.

### 2.9. BMI Assessment

To assess body mass index (BMI), participants used a bioimpedance body fat analyzer (BC-621, Tanita Co., Tokyo, Japan). Participants fasted for at least 8 h prior to measurement. Height and weight were recorded to calculate BMI using the formula BMI = weight (kg)/height (m^2^) [22].

### 2.10. Flow Mediated Dilation (FMD) before Consumption and at the End Period of Consumption the Product

To assess flow-mediated dilation (FMD) before and after mung bean protein powder consumption, healthy volunteers underwent baseline and post-6-week consumption tests. After fasting, brachial artery diameter was measured using Doppler ultrasound, followed by cuff inflation for five minutes to induce ischemia. The test was repeated after six weeks of daily consumption. The percentage change in diameter was calculated from ultrasonographic images obtained from Doppler ultrasound machine (GE Healthcare, Chicago, IL, USA) to evaluate the product’s effects on endothelial function and cardiovascular health.

### 2.11. Biochemical Assessments

Oxidative stress markers, including the malondialdehyde (MDA) level, and the activities of catalase (CAT) and glutathione peroxidase (GPx) were measured as previously mentioned [13,14]. In brief, the MDA was monitored using a thiobarbituric acid reactive substances assay (TBARS) method, and the CAT was assessed using the decomposition of H_2_O_2_ as an indicator. GSH-Px was also monitored by measuring the oxidation of NADH to NADP by the enzyme glutathione reductase during the oxidation of oxidized glutathione (GSSG), which is formed by the catalytic action of organic peroxide by GPx.

#### Serum Inflammatory Markers: TNF-α, IL-6, NF-kB, Nitrite, Nitrate, and NO

Serum levels of TNF-α, IL-6, NF-kB, nitrite, nitrate, and nitric oxide (NO) were determined using specific ELISA kits (Thermo Fisher Scientific, Waltham, MA, USA). The procedure followed the manufacturer’s instructions for each respective kit. Serum samples were prepared by centrifugation and stored at −80 °C until analysis. Each inflammatory marker was measured using specific assays, with absorbance readings taken at the appropriate wavelengths.

### 2.12. Statistical Analysis

All data are presented as mean ± standard error of the mean (SEM). Normality was assessed using the Kolmogorov–Smirnov test. A one-way analysis of variance (ANOVA) was conducted to compare group differences, followed by Tukey’s post hoc test. Statistical significance was set at a *p*-value of less than 0.05.

## 3. Results

### 3.1. Polyphenol and Flavonoids Contents of MB

The total phenolic compounds and flavonoids of MB were 450.47 ± 2.138 μg Gallic acid/g sample and 392.56 ± 0.663 μg quercetin/g, respectively, as shown in Table 1.

### 3.2. Amino Acid Profile of MB

Valine, isoleucine, and leucine, which are essential amino acids (EAAs) classified as branched-chain amino acids (BCAAs), were found in high quantities in mung bean protein, measuring 4790.4 mg, 3652.7 mg, and 9461.1 mg per 100 g of protein, respectively. Furthermore, a comparison of the total volumes of essential amino acids (EAAs) and non-essential amino acids (NEAAs) revealed that mung bean protein provides a beneficial amino acid profile that serves as a valuable plant-based protein source, although it does not match the amino acid levels typically found in whey protein concentrate, as shown in Table 2.

### 3.3. Biological Activities Profile of MB

The DPPH inhibition assay revealed an IC50 of 6.557 ± 0.026 mg/mL (Table 3), indicating a 20.32% inhibition rate, which suggests that the mung bean protein powder exhibits moderate antioxidant activity. The ABTS assay showed a lower IC50 of 4.950 ± 0.008 mg/mL with an 18.03% inhibition, confirming its ability to scavenge free radicals effectively. Additionally, the COX-2 inhibition assay indicated an IC50 of 5.622 ± 0.28 mg/mL and a 10.60% inhibition, reflecting the powder’s potential anti-inflammatory properties.

### 3.4. Composition and Biological Activities Profile of MB Functional Protein Drink and Placebo

Table 4 outlines the composition of the functional drink derived from mung bean protein and its placebo. The drink contains 3 g each of mung bean and soy powder (12 kcal each), with 10 g of mung bean protein concentrate adding 40 kcal. It also includes 3.8 g of maltodextrin (55.2 kcal), 5 g of brown sugar (20 kcal), and 0.4 g of salt (0 kcal). The total caloric content of the functional drink is 99.2 kcal, highlighting its nutritional advantages, particularly its higher protein content compared to the placebo.

The amino acid profile of the MB protein functional drink demonstrates a substantial advantage over the placebo, particularly in EAAs. Notably, the drink contains significantly higher levels of EAAs, such as leucine, isoleucine, and lysine. In total, the functional drink provides 585.15 mg/g of EAAs compared to just 34.79 mg/g in the placebo. Furthermore, the drink also offers a robust profile of NEAAs.

Table 5 highlights the biological activity of the functional drink and placebo formula, showing that the MB15 protein drink demonstrated superior activity across all parameters compared to both MB10 and the placebo. MB15 exhibited significantly lower IC50 values for DPPH (4.33 ± 0.01 mg/mL) and ABTS (2.35 ± 0.00 mg/mL) inhibition, indicating stronger antioxidant capacity compared to MB10 and placebo, with *p* < 0.05 when compared with MB10. Additionally, MB15 showed enhanced COX-2 inhibition (4.92 ± 0.01 mg/mL) and comparable ACE inhibition (4.87 ± 0.02 mg/mL) to MB10, both outperforming the placebo. These results suggest that the MB15 protein drink provides greater biological activity in terms of antioxidant and enzyme inhibition effects.

### 3.5. Data Characteristics of Subjects

Table 6 presents the general characteristics of the volunteers at baseline and after six weeks of supplementation with either a placebo or mung bean protein powder at two different dosages (MB10 and MB15). Overall, the data demonstrate that the baseline characteristics among the placebo and mung bean groups were comparable, with no significant differences across most parameters. After six weeks of supplementation, the measurements remained stable, suggesting no adverse effects or significant changes in general health metrics among the subjects.

### 3.6. Effect of the Functional Protein Derived from MB Drinks on Blood Oxidative Stress: Malondialdehyde (MDA) Levels, Catalase (CAT) Activity, and Glutathione Peroxidase (GPx) Activity

**Figure 2:**
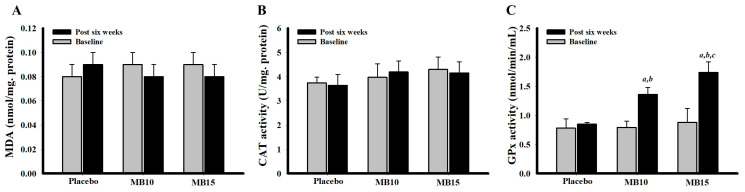
MDA levels (**A**) and antioxidant enzymes, catalase activity (**B**), and glutathione peroxidase (GPx) activity (**C**) of volunteers before and at 6 weeks after the intervention. Data are presented as mean ± SEM. (n = 9/group). *^a^ p* > 0.05 when compared with baseline, *^b^ p* < 0.05 when compared with placebo (post six weeks), and *^c^ p* < 0.05 when compared with MB10 post-intervention.

The oxidative stress parameters measured before and after six weeks of intervention show no significant changes in MDA levels across groups (Figure 2), indicating stable lipid peroxidation levels. Catalase (CAT) activity also remained consistent, with no significant differences observed. However, glutathione peroxidase (GPx) activity showed an increase in both MB10 and MB15 groups compared to baseline, with MB15 approaching statistical significance. These findings suggest that mung bean consumption may enhance certain antioxidant enzyme activities, contributing to improved oxidative stress profiles in healthy adults.

### 3.7. Effect of the Functional Protein Derived from MB Drinks on Inflammatory Markers: NF-kB, TNF-α, IL-6, Nitrite, Nitrate, and NO

**Figure 3:**
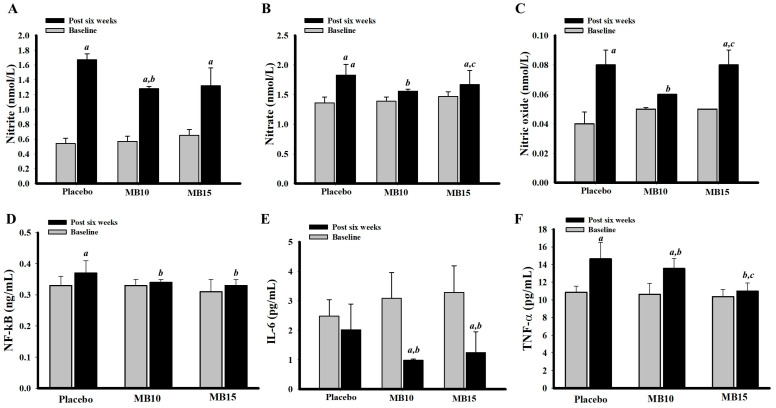
Inflammatory markers, including nitrite levels (**A**), nitrate (**B**), nitric oxide (**C**), NF-kB (**D**), IL-6 (**E**), and TNF-α (**F**) of volunteers before and at 6 weeks after the intervention. Data are presented as mean ± SEM. (n = 9/group). *^a^ p* > 0.05 when compared with baseline, *^b^ p* < 0.05 when compared with placebo (post six weeks), and *^c^ p* < 0.05 when compared with MB10 post-intervention.

Figure 3A–C presents the inflammatory levels of volunteers before and six weeks after the intervention, comparing a placebo group with two groups consuming MB functional drinks (MB10 and MB15). At baseline, there were no significant differences in nitrite, nitrate, or NO levels across the groups. However, after six weeks, the MB10 group demonstrated significant reductions in nitrite (1.28 ± 0.03 nmol, *p* = 0.010) and nitrate (1.39 ± 0.03 nmol, *p* = 0.010) compared to the placebo group, along with a decrease in NO levels (0.06 ± 0.00 nmol, *p* = 0.010). In contrast, the MB15 group did not show significant changes in any inflammatory markers when compared to the placebo.

Interestingly, after six weeks of consumption, the MB functional drinks (MB10 and MB15) significantly suppressed pro-inflammatory mediators, including NF-kB, TNF-α, and IL-6, compared to the placebo group (Figure 3D–F). These results suggest a potential anti-inflammatory effect associated with the intake of mung bean functional drinks.

### 3.8. Effect of the Functional Protein Derived from MB Drinks on FMD

**Figure 4:**
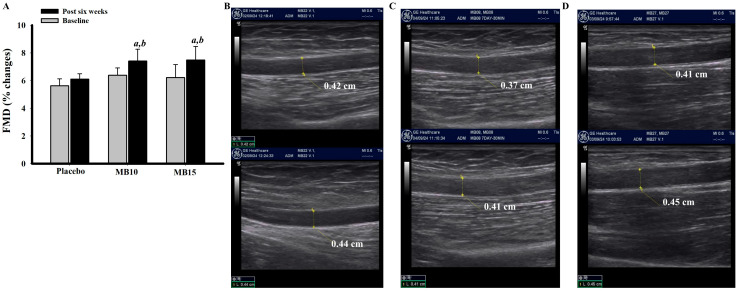
Flow-mediated dilation (FMD) of volunteers before and six weeks after the intervention (**A**). Representative ultrasonographic image elucidates brachial artery diameter before occlusion (upper panel) and reperfusion (lower panel) of placebo group (**B**), MB10 (**C**), and MB15 (**D**) performed after six weeks of intervention. Data are presented as mean ± SEM of %changes of baseline (n = 9/group). *^a^ p* > 0.05 when compared with baseline, *^b^ p* < 0.05 when compared with placebo (post six weeks).

The flow-mediated dilation status of volunteers indicated no significant differences among the groups prior to the intervention. However, after six weeks of consuming the assigned products, the MB10 and MB15 groups demonstrated a significant increase in the percentage change of flow-mediated dilation (Figure 4A). This measurement was obtained and calculated from ultrasonographic images of the diameter of the brachial artery before occlusion and reperfusion in each group.

## 4. Discussion

This study investigates the effects of six weeks of mung bean consumption on healthy middle-aged adults. The results indicate that mung beans exhibit significant in vitro antioxidative properties and are rich in essential amino acids (EAAs). Mung bean consumption enhances plasma antioxidant enzyme levels while reducing nitrite, nitrate, and NO levels, suggesting an anti-inflammatory effect. Additionally, there is a significant increase in the percentage change in brachial artery diameter following FMD in the MB-treated group compared to the placebo group, indicating cardiovascular protective effects.

The antioxidative effects of MB can be attributed to their high content of bioactive compounds, including polyphenols, flavonoids, and vitamins, which scavenge free radicals and mitigate oxidative stress. These compounds inhibit lipid peroxidation and modulate various antioxidant enzymes, thereby enhancing the body’s overall antioxidant capacity [23,24]. Furthermore, mung beans contain essential amino acids and other phytochemicals that contribute to their health-promoting properties. This complex mixture protects cells from damage, supports cardiovascular health, and reduces inflammation [25,26].

In vitro studies indicate that MB protein possesses significant amounts of EAAs and NEAAs, which play crucial roles in various physiological functions. EAAs, such as lysine and leucine, are vital for protein synthesis and muscle repair, while NEAAs, including glutamic acid and arginine, are important for metabolic pathways and may support endothelial function [16,26]. The balance of these amino acids in mung beans can enhance metabolic health and contribute to the beneficial effects observed in this study.

After six weeks, MB consumption significantly increases the activity of glutathione peroxidase, bolstering the body’s antioxidant defenses [24,27]. This enzyme plays a critical role in protecting cells from oxidative damage by decomposing hydrogen peroxide and reducing lipid peroxidation. The stability of malondialdehyde (MDA) levels during this period suggests that the antioxidant capacity induced by MB consumption effectively counterbalances oxidative stress without further lipid peroxidation [28].

Moreover, the reduction in nitrite, nitrate, and NO levels may stem from the modulation of nitric oxide synthase (NOS) activity by antioxidants and bioactive compounds in mung beans. The lack of significant changes in nitrite, nitrate, and NO levels in the MB15 group compared to the placebo may be due to a threshold effect, where the higher dosage does not yield additional benefits beyond a certain concentration of active compounds. The body may have already optimized its response to the bioactive components at lower doses, like those in the MB10 group. Additionally, the efficacy of specific bioactive compounds, such as antioxidants in mung beans, may diminish at higher concentrations, potentially leading to saturation effects that limit the expected anti-inflammatory benefits at the MB15 dosage. Mung beans also exhibit COX-2 inhibitory properties, which can reduce inflammation [29]. By inhibiting COX-2, MB may lower the production of pro-inflammatory mediators, contributing to the observed decreases in IL-6 levels and suggesting a potential for mitigating chronic inflammation [16,30]. This anti-inflammatory effect, coupled with their antioxidant capacity, may enhance cardiovascular health and reduce the risk of diseases associated with inflammation.

Additionally, MB demonstrates an ability to suppress TNF-α levels and inhibit the NF-κB signaling pathway, both of which are critical in mediating inflammatory responses. The downregulation of TNF-α, a key pro-inflammatory cytokine, in conjunction with NF-κB inhibition, underscores the potential of mung beans to alleviate systemic inflammation and promote overall health [31,32].

Furthermore, mung beans exhibit ACE inhibition, further promoting vascular function. The ACE-inhibitory action can help regulate blood pressure and improve endothelial function [33]. While NO is essential for vasodilation and immune responses, excessive production can lead to inflammatory processes. The decrease in these markers suggests that mung beans may effectively mitigate inflammatory pathways. This dual action, combining ACE inhibition and antioxidant effects, promotes cardiovascular health by improving endothelial function and reducing the risk of chronic diseases [34].

The influence of MB consumption on FMD, even with reduced NO levels, involves complex mechanisms. MB may enhance endothelial function and vascular health through improved antioxidant capacity [18]. While the decrease in NO may indicate a balanced modulation of NOS activity, it also suggests enhanced sensitivity of blood vessels to existing NO, improving vasodilation and FMD. This overall decrease in oxidative stress and inflammatory markers, along with improved endothelial function, supports the cardiovascular benefits of mung beans even with lower NO levels.

## 5. Conclusions

In conclusion, this study provides evidence that the consumption of the functional protein derived from MB, rich in AAs), for six weeks significantly benefits healthy middle-aged adults by enhancing antioxidant enzyme activity and suppressing pro-inflammatory mediators. The observed increase in brachial artery diameter following flow-mediated dilation indicates an improvement in cardiovascular health. Collectively, these findings position mung beans as a functional food that promotes cardiovascular protection while mitigating oxidative stress and inflammation.

## Figures and Tables

**Table 1:** Phytochemical contents: total phenolic content and total flavonoid content of a functional protein derived from mung MB.

Phytochemical Contents	A Functional Protein Derived from MB
Total phenolic content	450.47 ± 2.138 μg Gallic acid/g
Total flavonoid content	392.56 ± 0.663 μg Quercetin/g

**Table 2:** Amino acid profiles of a functional protein derived from MB, soy, and whey protein concentrate in mg/100 g protein.

Amino Acid	Functional Protein Derived from MB (mg/g)	Soy Protein Concentrate (mg/g)	Whey Protein Concentrate (mg/g)
EAAs			
Threonine	31.557	24.740	72.000
Methionine	12.036	8.140	19.000
Phenylalanine	70.010	32.780	33.000
Histidine	96.407	15.780	22.000
Lysine	71.856	39.290	96.000
Valine	47.904	30.640	58.000
Isoleucine	36.527	29.420	58.000
Leucine	94.611	49.170	102.390
Tryptophan	6.826	8.350	21.000
Arginine	117.365	46.420	21.000
Total EAAs	585.150	284.730	502.390
NEAAs			
Serine	60.480	33.690	47.000
Glycine	17.844	26.880	18.000
Glutamic acid	144.311	120.130	167.000
Proline	58.683	32.980	58.000
Cysteine	0.916	8.860	20.890
Alanine	42.695	26.770	49.000
Tyrosine	27.066	23.010	18.000
Aspartic acid	131.747	72.490	108.000
Total NEAAs	483.730	344.810	485.890

**Table 3:** Biological activities of a functional protein powder derived from MB.

Parameters	A Functional Protein Powder Derived from MB	
	IC50 (mg/mL)	% Inhibition (1 mg/mL of MB)
DPPH inhibition	6.557 ± 0.026	20.32 ± 0.15
ABTS inhibition	4.950 ± 0.008	18.03 ± 0.15
COX-2 inhibition	5.622 ± 0.28	10.60 ± 0.06
ACE inhibition	4.061 ± 0.005	13.72 ± 0.28

**Table 4:** Composition of the functional drink and placebo formula with adjusted calories.

Ingredient/Amino Acid	MB10 Protein Drink (g/mg)	Placebo (g/mg)	Calories (kcal)
Ingredients			
Mung bean powder	3.00	3.00	12
Soy powder	3.00	3.00	12
Mung bean protein concentrate	10.00	-	40
Maltodextrin	3.80	13.80	55.2
Brown sugar	5.00	5.00	20
Salt	0.40	0.40	0
Total	25.20	25.20	99.2 kcal
Amino acids			
Tryptophan	6.83	1.12	
Threonine	31.56	3.14	
Isoleucine	36.53	4.25	
Leucine	94.61	6.78	
Lysine	71.86	5.33	
Methionine	12.04	1.13	
Phenylalanine	70.01	4.59	
Valine	47.90	4.10	
Histidine	96.41	2.30	
Total EAAs	585.15	34.79	
Arginine	117.37	6.67	
Cystine	0.92	1.05	
Tyrosine	27.07	3.22	
Alanine	42.70	3.59	
Aspartic acid	131.74	10.20	
Glutamic acid	144.31	17.50	
Glycine	36.00	3.60	
Proline	58.68	4.96	
Serine	60.48	4.59	
Total NEAAs	603.70	55.79	

**Table 5:** The biological activity of the functional drink and placebo formula.

Parameters	Placebo	MB10 Protein Drink	MB15 Protein Drink
DPPH inhibition (IC50: mg/mL)	7.75 ± 0.01	5.68 ± 0.02 *^a^*	4.33 ± 0.01 *^a,b^*
ABTS inhibition (IC50: mg/mL)	4.71 ± 0.03	3.32 ± 0.01 *^a^*	2.35 ± 0.00 *^a,b^*
COX-2 inhibition (IC50: mg/mL)	6.34 ± 0.02	5.35 ± 0.02 *^a^*	4.92 ± 0.01 *^a,b^*
ACE inhibition (IC50: mg/mL)	6.62 ± 0.02	4.94 ± 0.02 *^a^*	4.87 ± 0.02 *^a^*

*^a^ p* > 0.05 when compared with placebo formula, *^b^ p* < 0.05 when compared with MB10 protein drink.

**Table 6:** General characteristics of volunteers.

**General Characteristics**	**Baseline**		
	**Placebo (n = 9)**	**MB10 (n = 9)**	**MB15 (n = 9)**
Age (year)	49.44 ± 1.04	50.22 ± 1.74 (*p* = 0.929)	51.67 ± 1.68 (*p* = 0.307)
Gender (male/female)	0/9	1/8	1/8
Blood temperature (°C)	36.54 ± 0.06	36.61 ± 0.06 (*p* = 0.407)	36.56 ± 0.05 (*p* = 0.889)
Heart rate (beats/min)	71.33 ± 2.58	67.33 ± 3.36 (*p* = 0.353)	70.89 ± 2.96 (*p* = 0.917)
Respiratory rate (breaths/min)	17.22 ± 0.22	16.78 ± 0.28 (*p* = 0.219)	17.33 ± 0.24 (*p* = 0.696)
Systolic blood pressure (mmHg)	113.67 ± 3.14	110.56 ± 3.86 (*p* = 0.554)	109.22 ± 3.95 (*p* = 0.400)
Diastolic blood pressure (mmHg)	74.22 ± 2.37	69.22 ± 2.19 (*p* = 0.122)	71.67 ± 2.05 (*p* = 0.421)
Body weight (kg)	56.71 ± 2.88	53.32 ± 1.79 (*p* = 0.270)	52.77 ± 1.43 (*p* = 0.201)
Body height (cm)	158.11 ± 1.39	155.56 ± 2.32 (*p* = 0.348)	156.56 ± 1.83 (*p* = 0.565)
BMI (kg/m^2^)	22.59 ± 0.87	22.06 ± 0.67 (*p* = 0.624)	21.59 ± 0.75 (*p* = 0.363)
**General Characteristics**	**After 6 Weeks Consume**		
	**Placebo (n = 9)**	**MB10 (n = 9)**	**MB15 (n = 9)**
Age (year)	49.44 ± 1.04	50.22 ± 1.74 (*p* = 0.929)	51.67 ± 1.68 (*p* = 0.307)
Gender (male/female)	0/9	1/8	1/8
Blood temperature (°C)	36.58 ± 0.05	36.61 ± 0.03 (*p* = 0.703)	36.59 ± 0.07 (*p* = 0.963)
Heart rate (beats/min)	69.56 ± 2.48	69.56 ± 3.13 (*p* = 0.790)	70.11 ± 2.08 (*p* = 0.929)
Respiratory rate (breaths/min)	17.33 ± 0.29	17.33 ± 0.17 (*p* = 0.771)	17.44 ± 0.18 (*p* = 0.961)
Systolic blood pressure (mmHg)	110.22 ± 3.40	106.00 ± 2.94 (*p* = 0.378)	107.33 ± 3.60 (*p* = 0.545)
Diastolic blood pressure (mmHg)	69.22 ± 2.53	65.78 ± 2.44 (*p* = 0.306)	68.67 ± 1.99 (*p* = 0.868)
Body weight (kg)	56.60 ± 2.88	52.99 ± 1.94 (*p* = 0.258)	52.92 ± 1.58 (*p* = 0.250)
Body height (cm)	158.11 ± 1.39	155.56 ± 2.32 (*p* = 0.348)	156.56 ± 1.83 (*p* = 0.565)
BMI (kg/m^2^)	22.55 ± 0.87	21.92 ± 0.75 (*p* = 0.582)	21.65 ± 0.78 (*p* = 0.433)

BMI; Body mass index, °C; degree Celsius, mmHg; millimeter of mercury, min; minutes, kg; kilogram, cm; centimeter, m^2^; square meter. Data are presented as mean ± SEM. (n = 9/group).

## Data Availability

The original contributions presented in the study are included in the article, further inquiries can be directed to the corresponding author.
